# Nutrients and Dietary Approaches in Patients with Type 2 Diabetes Mellitus and Cardiovascular Disease: A Narrative Review

**DOI:** 10.3390/nu13114150

**Published:** 2021-11-19

**Authors:** Carlos Jiménez-Cortegana, Pedro Iglesias, Josep Ribalta, Teresa Vilariño-García, Laura Montañez, Francisco Arrieta, Manuel Aguilar, Santiago Durán, Juan C. Obaya, Antonio Becerra, Juan Pedro-Botet, Víctor Sánchez-Margalet, on behalf of the Cardiovascular Disease Working Group of the Spanish Society of Diabetes (SED)

**Affiliations:** 1 Department of Medical Biochemistry and Molecular Biology and Immunology, Virgen Macarena University Hospital, University of Seville, Av. Dr. Fedriani 3, 41009 Seville, Spain; cjcortegana@gmail.com (C.J.-C.); tvgarcia@us.es (T.V.-G.); 2 Endocrinology and Nutrition Service, Puerta de Hierro University Hospital, Majadahonda, 28034 Madrid, Spain; piglo65@gmail.com; 3 Departament de Medicina i Cirurgia, University Rovira i Vigili, IISPV, CIBERDEM, 43007 Tarragona, Spain; josep.ribalta@urv.cat; 4 Endocrinology and Nutrition Service, Ramón y Cajal University Hospital, 28034 Madrid, Spain; laura.montanez@salud.madrid.org (L.M.); arri68@hotmail.com (F.A.); 5 Endocrinology and Nutrition Service, University Hospital “Puerta del Mar”, Instituto de Investigación e Innovación en Ciencias Biomédicas de la Provincia de Cádiz (INiBICA), Cádiz University (UCA), 11001 Cádiz, Spain; manuelaguilardiosdado@gmail.com; 6 Endodiabesidad Clínica Durán & Asociados, 41009 Seville, Spain; sduran@duransanz.com; 7 CHOPERA Health Center, Alcobendas Primary Care, 28034 Madrid, Spain; juancarlosobaya@yahoo.es; 8 Department of Nutrition, University of Alcalá, 28034 Madrid, Spain; becerrafernandez@gmail.com; 9 Lipids and Cardiovascular Risk Unit, Hospital del Mar, Autonomous University of Barcelona, 08003 Barcelona, Spain; jpedrobotet@psmar.cat

**Keywords:** type 2 diabetes, caridovascular risk, nutrients, diets

## Abstract

Cardiovascular disease (CVD) is the most common cause of morbidity and mortality in developed countries. The prevalence of CVD is much higher in patients with type 2 diabetes mellitus (T2DM), who may benefit from lifestyle changes, which include adapted diets. In this review, we provide the role of different groups of nutrients in patients with T2DM and CVD, as well as dietary approaches that have been associated with better and worse outcomes in those patients. Many different diets and supplements have proved to be beneficial in T2DM and CVD, but further studies, guidelines, and dietary recommendations are particularly required for patients with both diseases.

## 1. Introduction

According to the Global Burden Disease Study 2019, cardiovascular disease (CVD) is the most common cause of morbidity and mortality in developed countries since it leads to almost 20 million deaths per year worldwide with a prevalence over 500 million people [1]. Specifically, CVD underlies around 300 deaths per 100,000 population per year in the United States and 500 deaths per 100,000 population per year in China [2,3], and more than 4 million deaths per year in Europe, resulting in 45% of all deaths in this continent [4]. However, 90% of CVD cases could be prevented [5]. Atherosclerosis is the main cause of CVD [6] and comprises a set of diseases that share risk factors and strategies for primary and secondary prevention [3,7] such as coronary heart disease (CHD) [8] or different ischemic vascular events [9].

Although the exact cause of CVD remains unknown, some risk factors raise the probability of developing the disease, including hypertension [10], smoking [11], family history [12], gender [13], overweight and obesity [14], sedentarism [15], hypercholesterolemia [16], or diabetes mellitus (DM) [17], among others [18]. Specifically, DM is a heterogeneous disorder characterized by hyperglycemia, insulin resistance (IR) and insulin deficiency (ID). Most of the risk factors that foster DM also promote CVD. The relationship between both diseases involves altered signaling pathways, which ultimately promote chronic inflammation, elevated glucose levels, dyslipidemia, IR, and hyperglycemia [19,20]. Furthermore, meta-analyses and systematic reviews confirmed that DM increases the risk of CVD, which is the highest in patients with existing vascular disease, proteinuria, or renal disease, and the lowest in those newly diagnosed [21]. Thus, it is not surprising that CVD is the leading cause of morbidity and mortality among individuals with DM [22]. Changes in lifestyle, such as the intake of ultra-processed foods (UPFs) or sedentary habits, have been associated with worse outcomes. For example, the Moli-sani Study correlated high intakes of UPFs with increased risks of CVD mortality, death from ischemic heart disease or cerebrovascular disease, and all-cause mortality [23].

The current situation of cardiometabolic patients could probably be improved or even reversed by nutritional intervention, which consists of an adequate nutrition by adapting dietary patterns and reducing calorie intake [24] as it has been successfully proven in some conditions such as cancer [25], neurodegenerative diseases [26], or heart failure [27]. However, the role of nutritional intervention in patients with CVD and T2DM is scant [28,29,30,31] and should be well-described and updated from time to time since it would allow learning to identify each food group correctly as well as establishing better diets as a fundamental part of the treatment for those patients. For these reasons, and due to the link that obesity has with multiple metabolic dysfunctions that increase the risk of CVD, the aim of this review was to examine the importance of nutrition in patients with both type 2 diabetes mellitus (T2DM) and CVD.

## 2. Dietary Nutrients for the Management of Patients with CVD and DM

**Figure 1:**
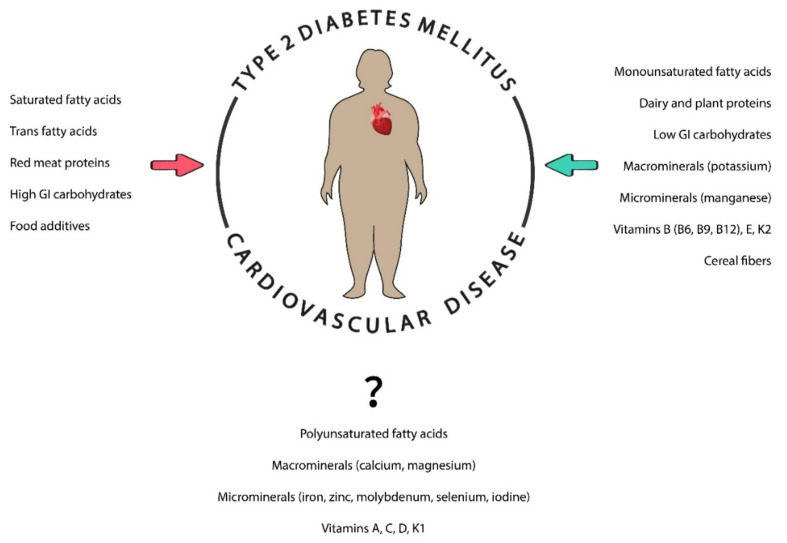
The role of different nutrients in individuals with diabetes and cardiovascular diseases. Nutrients can be beneficial (green arrow) or prejudicial (red arrow) in these patients. GI: glycemic index.

The importance of nutrition in preventing CVD and DM individually is well established. Dietary management of these diseases has been focused on the study of macronutrients (carbohydrates, fats, proteins, macrominerals, and water), micronutrients (vitamins and other minerals), and other nutrients (fiber, food additives, and dietary supplements) to control the balance between energy expenditure and calorie intake. It is necessary to highlight the importance of food quality over food quantity by having dietary patterns rich in whole grains, fruit, vegetables, nuts, legumes, fish, or vegetable oils and poor in processed meats, refined grains, refined carbohydrates, and salt [32,33]. In order to achieve these behavioral patterns, many recommendations and guidelines have been developed and implemented while also considering other points such as personal preferences (e.g., tradition, culture, religion, or economics) and metabolic goals [34,35,36]. In addition, different genetic polymorphisms have been found in obese patients with CVD who used the same dietary patterns, and some gene variants have been associated with different responses to specific nutrients, suggesting that knowledge of the individual’s genetic backgrounds could also be essential [37]. The contribution of different nutrients in patients with T2DM and CVD is shown in Figure 1.

### 2.1. Macronutrients

#### 2.1.1. Fats

Disorders of lipid metabolism (e.g., dyslipidemias) are widely known to play a key role in atherosclerosis and T2DM [38,39,40,41], mainly due to chronic inflammation, which is considered as one of the possible links between both diseases [19]. A systematic review with 57,079 participants demonstrated that patients with low fat intake had small but stable effect by reducing weight, body mass index (BMI), waist circumference, and percentage of body fat compared with those who had higher fat intake [42]. However, it is necessary to specify that there are various types of fats that, based on their chemical structures and physical properties (saturated, trans, monounsaturated, and polyunsaturated), exert different effects on plasma lipids and lipoproteins.

Saturated fatty acids (SFAs) present different chain lengths, with lauric, myristic, palmitic, and stearic acids as the major ones. SFAs can be found in both vegetable (e.g., coconut, palm oil) and animal fats (e.g., meat, butter, and cheese). Dietary guidelines generally recommend a low-SFA diet (up to 10% of total calories) [38,39,43]. Dietary SFAs strongly raise total and low-density lipoproteins (LDL) cholesterol levels in blood. In this respect, excess fatty acids, together with IR and hyperglycemia, disrupt protein kinase C signaling, increase oxidative stress, advance glycation end products, and promote the so-called atherogenic or diabetic dyslipidemia, characterized by increased triglycerides, low high-density lipoproteins (HDL)-cholesterol, and an increased number of smaller LDL particles [44], which could result in vascular inflammation, vasoconstriction, thrombosis and atherogenesis [45], thereby enhancing the risk of CVD and mortality in T2DM individuals [46,47,48,49]. In fact, a small reduction intake of SFAs has been associated with a reduction in cardiovascular risk [50]. By contrast, other studies found that SFAs have been uncorrelated with all-cause mortality, CVD, coronary heart disease (CHD), the major contributor to total mortality, ischemic stroke, or T2DM [51]. Furthermore, the Cardiovascular Health Study found very-long-chain SFAs were associated with a lower risk of diabetes, suggesting the need to distinguish the effects of different circulating fatty acids [52].

Monounsaturated fatty acids (MUFAs) are found in olive oil, nuts, corn, and avocados. MUFAs are closely related to SFAs since MUFAs can also be found in some foods with high SFA content [28]. Many studies have concluded that consuming diets high in MUFAs (e.g., the Mediterranean diet), can improve metabolic risk factors in patients with obesity, T2DM, and CVD [53,54,55], since MUFAs could favorably influence plasma lipid and lipoprotein concentrations, which are predictors of CHD events in both non-diabetic and diabetic individuals [49].

Polyunsaturated fatty acids (PUFAs) may be beneficial in cardiometabolic diseases. Two main types of PUFAs exist according to their chemical structure: omega-3 (Ω-3 PUFAs) and omega-6 (Ω-6 PUFAs). Ω-3 PUFAs include docosahexaenoic acid (DHA), α-linolenic acid (ALA) and eicosapentaenoic acid (EPA), and can be found in salmon and tuna. Ω-6 PUFAs are found in plants, such as sunflower seeds or olive oil, and include arachidonic acid, linoleic acid, and γ-linolenic acid. Although PUFAs appeared to prevent cardiovascular events, they are prone to oxidation and its role remains controversial in CVD [56,57,58,59]. The most extensive systematic analysis of Ω-3 PUFAs in CVD showed moderate- to low-certainty evidence of their beneficial role in reducing the risk of CHD events, CHD mortality and serum triglycerides [60]. Unclear results have also been obtained in DM [61,62,63]. In T2DM patients with CVD, data related to the role of PUFAs are inconsistent [64,65,66] owing, at least in part, to different supplementation dosages and duration [65,67]. High doses of Ω-3 PUFAs did not improve metabolic status in T2DM patients with CVD [65]. The role of Ω-6 PUFAs also remain unclear in both diseases. Ω-6 PUFAs have been associated with hyperinsulinemia rather than a protective role in CVD [66], and poorly correlated with reduced risks of CHD, CHD mortality and stroke [68]. In addition, a meta-analysis carried out by Al-Khundary et al. (2015) did not find sufficient evidence to associate the levels of Ω-6 PUFAs with cardiovascular risks [69].

Trans-fatty acids (TFAs) are found in snacks, baked, and or fried foods, such as chocolates, biscuits, burgers, pizzas, and French fries, and their intake has significantly increased owing to food industrialization. TFAs can also be found in conjunction with saturated fats in processed foods, whole milk, butter, margarine, and cheese [34]. TFAs are produced by partial hydrogenation, in which hydrogen atoms are added to cis-unsaturated fat, thus eliminating the double bonds between carbon atoms and rendering them saturated [70]; however, some of those cis bonds are converted into trans bonds, resulting in TFAs instead of SFAs. The advantage of hydrogenation is that it extends shelf life by making fats less prone to oxidation as well as rendering the product more suitable for frying, since this process increases the fat’s melting point [28]. The consumption of TFAs has been shown to influence CVD risk factors, including DM, by causing endothelial dysfunction [71], raising serum LDL cholesterol or triglyceride levels, or by lowering HDL cholesterol levels or LDL particle size [72]. TFAs also promote CHD and CHD mortality [51] as well as systemic inflammation in women with high BMI, being associated with high tumor necrosis factor (TNF) activity and increased IL-6 and C-reactive protein levels [73]. In addition, both trans-palmitoleic acid and pentadecanoic acid increased in T2DM patients with a high-fat diet compared with their low-fat diet counterparts and controls, being associated with changes in triglycerides, total cholesterol, and very-low-density lipoprotein cholesterol (VLDL-C) [74], although no changes were found on glycated hemoglobin (HbA1c), body weight, body composition, lipid profile, and blood pressure of those patients, irrespective of the dietary fat content in those patients [75]. However, the classification of different TFAs could be needed, since ruminant TFAs may play an important cardioprotective role [76]. Ruminant TFAs differ from industrial TFAs and are found in foods derived from grass-grazing sheep and cattle such as their meat or dairy products; however, their beneficial role remains to be completely elucidated [77,78].

#### 2.1.2. Proteins

Proteins are found in different foods and amino acids determined their quality. Animals provide proteins of high biological value, which are mainly found in foods of animal origin (e.g., meat, fish, shellfish, dairy products, and eggs), whereas plants, legumes, grains, nuts, seeds, and vegetables provide low biological value proteins.

Proteins have been demonstrated to be essential for optimal growth, development, and health, and could reduce overweight and obesity, although their overconsumption may result in digestive, renal, and vascular abnormalities [79,80]. Protein intake for CVD patients remains controversial [80,81,82]. Some studies have shown that red meat increases the risk of CHD and should be reduced or substituted by other protein sources such as dairy products, which have been inversely correlated with hypertension, obesity and IR [83,84]. A high-protein intake also remains inconclusive for DM patients [80] since it has been associated with increased cardiometabolic disease risk [85], whereas a higher intake of plant protein is associated with a lower risk of DM [86]. Protein intake can also be beneficial in improving glycemic control [87] since proteins do not raise blood glucose levels [28], or their increment is relatively small [88]. On the other hand, protein intake should be decreased in the case of kidney disease, unless the patient is in dialysis.

#### 2.1.3. Carbohydrates

Different studies have suggested that atherosclerosis and T2DM are mainly driven by hyperglycemia due to IR, thus promoting inflammatory mediators, cellular damage, or other immune disruptions which could result in susceptibility to infections [89,90]. Different factors could alter blood glucose levels, including the types and total amount of carbohydrates as well as food particle size, pH, or the content of proteins, fats, and fibers, which also affect the glycemic index (GI) [91]. GI measures the percentage of the area under the curve with respect to 2-h blood glucose after the ingestion of a test diet compared with a standard diet (usually bread or glucose) [92]. In this respect, foods with carbohydrates that quickly break down during digestion and are rapidly absorbed into the bloodstream, raising both blood glucose levels and insulin responses, have a high GI (e.g., glucose, breakfast cereals, white wheat bread, watermelon, and mashed potatoes). By contrast, low GI is characterized by slow breakdown during digestion and slow assimilation, thus having a lesser impact on blood glucose levels and insulin responses (e.g., legumes, fructose, soy milk, apples, oranges, and barley) [91,93]. Another parameter, glycemic load (GL), is the product of carbohydrate quantity by GI, and indicates glucose availability for energy or storage after eating [94]. It is established that lower GI and GL confer benefits to prevent and manage CVD and DM [95]. In this regard, GI and GL have been positively associated with a significant increased risk of CVD and DM in women [96,97,98,99]. In the general population, it was demonstrated that both high and low percentages of carbohydrate diets were associated with increased mortality, with the lowest risk reported at 50–55% energy from carbohydrate [100]. Moreover, recent studies support that low carbohydrate diets (LCDs) may improve many renal and cardiovascular risk factors in patients with T2DM [101].

#### 2.1.4. Macrominerals

Macrominerals, such as calcium, phosphorus, magnesium, potassium, and sodium, are mainly found in fruits and vegetables, and are required in larger amounts. To date, there has been much controversy related to macromineral intake for both CHD and T2DM patients. Many studies have demonstrated that water calcium has a protective role against cardiovascular events [98,99,100,101,102,103,104,105], while others found no relationship between calcium and CVD [106] or T2DM [107]. Other macrominerals, such as phosphorus, potassium, and magnesium, are involved in different components of the metabolic syndrome (e.g., insulin secretion) [108]. In line with this notion, magnesium could exert an important influence on the pathogenesis of CVD since the kidney is a key regulator of magnesium homeostasis and kidney-related disorders can potentially lead to both magnesium depletion and overload [109,110]. By contrast, low magnesium levels could result in impaired insulin action as well as an altered cellular glucose transport, which promotes peripheral IR in T2DM [111], and its dietary supplementation may reduce the risk of T2DM-associated CVD due to a favorable effect on HDL, LDL, fasting plasma glucose, C-reactive protein (CRP), and insulin [112,113]. The beneficial role of potassium seems clear since its intake and a lower sodium-to-potassium ratio are associated with a lower risk of CVD, CHD, and hypertension, thereby improving glucose control and limiting the risk of diabetes [114,115,116,117]. On the same lines, epidemiologic studies suggest that low dietary potassium intake or serum potassium levels are associated with a higher risk of IR or T2DM [118].

#### 2.1.5. Water

Water constitutes another essential part of macronutrients and can be found in drinking water form or solid foods such as fruit and vegetables. Although water intake has not been studied in patients with both T2DM and CVD, it has been correlated with a lower risk of T2DM [119]. Chemical composition of water, which depends on the concentration of minerals and other substances in tap water, may be prejudicial in CVD patients, even though hard water may be beneficial. In this respect, it has been shown that long-term exposure to low levels of lead and arsenic may increase the risk of coronary events [120,121]. By contrast, calcium and magnesium from water may be beneficial, as some studies reported that a low magnesium intake could raise the risk of CVD, T2DM, and IR [111,122]. Supplementation with hydrogen-rich water may also be beneficial in the prevention of T2DM and IR since it was proven to normalize oral glucose tolerance tests and has been associated with decreased serum concentrations of oxidized LDL and free fatty acids as well as higher plasma levels of adiponectin and extracellular-superoxide dismutase [123].

### 2.2. Micronutrients

#### 2.2.1. Microminerals

Microminerals (iron, copper, zinc, manganese, molybdenum, iodine, fluorine, cobalt, and selenium) are necessary for normal functioning of the body in low concentrations. They are found in a wide variety of foods of animal and plant origin, including meat, fish, dairy products, fruit, and vegetables.

The role of iron remains controversial in DM and CVD. By analyzing diverse ethnic and geographic populations, epidemiologic studies provided strong evidence that iron intake contributes to oxidative damage as well as ID and IR, which is associated with DM, and to the development of atherosclerotic plaques, which increases the risk of CVD [124]. In addition, The Cohort on Diabetes and Atherosclerosis Maastricht study observed associations between iron metabolism (ferritin, transferrin, serum iron, and non-transferrin-bound iron) with adipocyte IR and T2DM [125]. Heme iron intake was associated with a high risk of developing T2DM in subjects with high cardiovascular risk [126]. However, low iron status was also associated with CVD risk in T2DM patients [127].

The roles of zinc and copper also remain controversial, possibly due to micromineral disturbances. Zinc is a trace element with a potent immunoregulatory role and has shown insulin-like action both in vitro and in vivo experiments [128]. A meta-analysis suggested that higher levels of copper were present in DM patients compared with healthy individuals [129], while both in vitro and in vivo studies showed that zinc had beneficial effects in the disease [128]. In CHD, one study showed mortality to be positively associated with copper intake in women and men, and inversely correlated with zinc intake in men but not in women [130]. Other studies concluded that zinc intake could reduce the risk of T2DM by 13%, whereas elevated serum or plasma zinc levels were associated with a 64% increased risk of T2DM [131]. It has also been suggested that zinc deficiency is negatively correlated with IL-6, promoting inflammation, T2DM, and atherosclerosis [132].

Manganese intake seems beneficial. A strong inverse relationship between its intake and the risk of T2DM has been reported in women but not men [133], which could be mediated by inflammatory biomarkers in postmenopausal women [134]. Urinary manganese has also been inversely correlated with systolic and diastolic blood pressure, thus protecting against hypertension [135]. However, long manganese exposure could result in acute CVD [136].

More studies are required on other microminerals since they have been less considered and results remain controversial. Serum molybdenum levels have been associated with T2DM [137], whereas the relationship between tungsten and CVD incidence has proved to be molybdenum-dependent, which underlines the promising role of molybdenum exposure in the disease [138]. Selenium and iodine may be implicated in DM, although results of epidemiologic studies and animal experiments are unclear [139]. Some reviews suggested that low urine iodine concentrations may be associated with coronary artery disease [140] and that optimum selenium intake could prevent atherosclerosis [141]; however, those results need to be confirmed by prospective studies.

Chromium is classified as an essential trace element, important in carbohydrate, lipid, and protein metabolism, because when it is deficient, it might lead to glucose intolerance and IR [142]. Chromium has been suggested in both in vitro and small in vivo human studies that has potentially beneficial effects in T2DM. It is important to highlight that a substantial proportion of the population is taking over the counter supplements that include chromium, and at a population level, many specifically are using chromium by name in their supplements. Clinical safety and efficacy data for chromium supplementation for improved insulin sensitivity and glycemic lowering is lacking. To support widespread use for diabetes treatment or prevention, and given the prevalence of current use, clinical trials adequately and aimed at evaluating safety and efficacy may be warranted [143].

#### 2.2.2. Vitamins

Vitamins are another essential part of nutrition and are also required in low concentrations. They are found in a large variety of foods, including meat, fish, fruit, vegetables, dairy products, and cereals. The role of vitamins in patients with both DM and CVD has been little investigated. For example, in this type of patient, vitamin E intake proved to play a cardioprotective role, since it lowers the risk of cardiovascular complications, morbidity and mortality [144,145,146], which may be mediated by an improvement in HDL functionality [147]. In addition, vitamin E could improve the outcome of cardiovascular events in patients with CVD or diabetes [148]. Vitamin C intake improved endothelial function in patients with T2DM and coronary artery disease [149]. However, other studies suggested there was no relationship between vitamin C supplementation and improved CVD risk factor status in diabetic individuals [150,151], which could lead to increased mortality from CVD in postmenopausal women with DM [151].

Other types of vitamins have been individually studied in DM and CVD. In this respect, the maintenance of serum vitamin D levels may be beneficial in both diabetic and cardiovascular conditions [152], since high vitamin D levels have been shown to be inversely correlated with CVD [153] and its deficiency has been associated with vascular dysfunction, hypertension, hyperlipidemia, and T2DM [154,155]. This can be explained because vitamin D regulates a variety of genes involved in important cardiovascular processes, such as cell proliferation, apoptosis, or oxidative stress, and its receptors have been found in cardiomyocytes, arterial wall cells, and immune cells [156]. By contrast, vitamin D supplementation did not produce clear improvements in blood pressure and insulin sensitivity in CVD patients [154,157], thus suggesting that the role of vitamin D in CVD, diabetes, or other cardiometabolic diseases could be inconclusive [158].

Retinol (vitamin A) has been both positively and inversely correlated with CVD and its role in T2DM remains unclear [159]. However, retinol-binding protein 4 contributes to IR and atherosclerosis in T2DM and could be used as an early predictor of CVD [160,161]. The role of vitamin K needs to be clarified since it was associated with a reduced risk of CVD in Mediterranean individuals [162] and menaquinone intake (vitamin K2) could be essential for CHD prevention [163,164]; however, no evidence exists of the role of phylloquinone (vitamin K1) in cardiometabolic disorders, including CVD and diabetes [164]. In addition, vitamin K-dependent protein activity was associated with incident ischemic cardiovascular events [165].

By contrast, the role of vitamin B is quite promising in both DM and CVD. There are several types of vitamin B, although folate or folic acid (vitamin B9), vitamin B6, and vitamin B12 have been the most studied. In this respect, folate and vitamin B12 levels were not associated with the risk of cardiovascular disorders, including stroke, coronary artery disease, myocardial infarction, or peripheral arterial disease [166]. Folate intake has also been inversely associated with DM, being even more beneficial than both vitamins B6 and B12 [167]. Furthermore, high-dose vitamin B supplementation (which included folic acid, vitamin B6, and vitamin B12) significantly slowed the progression of early-stage subclinical atherosclerosis [168]. It is important to note that low folate and B12 levels, especially in DM taking metformin, raise homocysteine levels, and this is a cardiovascular risk factor. The determination of folate and B12 is recommended in DM patients [169].

### 2.3. Other Nutrients

Dietary fibers (e.g., arabinoxylan, β-glucan, pectin, bran, and resistant starches) are non-digestible carbohydrates owing to lack of the required digestive enzymes [163,164]. Fiber intake has been shown to play an important role in human health, including lowering the risk of many types of cancer, precancer lesions, and cardiometabolic diseases such as CVD, CVD mortality, obesity, and DM [170,171,172]. However, only cereal fibers appear to improve IR and protect against T2DM compared with other types of dietary fibers such as fruit fibers [173,174]. The mechanisms remain unclear, but could be attributed to processes such as increased antioxidants, vitamins, minerals, short-chain fatty acid production, reduced calorie intake, prevention of dietary protein absorption, and modulation of the amino acid metabolic signature [170,174].

However, additives (e.g., glutamates, emulsifiers, and sulfites), mainly found in processed foods, have been suggested to be prejudicial. According to the NutriNet-Santé prospective cohort study conducted by Srour et al. (2019), a greater intake of ultra-processed foods was associated with higher risks of CVD and CHD. They also stated that several adverse effects of additives were observed in experimental studies on animal or cellular models [175]. For example, monosodium glutamate from sauces or noodles increased oxidative stress in mice [176], sulfites from sauces containing vinegar were harmful for rat hearts [177], and acesulfame K was able to promote atherosclerosis in cellular models [178].

Dietary supplements, whose industry has benefits over $100 billion globally, are believed by consumers to be necessary for a healthy diet or disease treatment. Some supplements such as fiber, selenium, and zinc represent an improvement in T2DM by most of studies, but others such as phosphorus, pantothenic acid, calcium, magnesium, glutamine, isoleucine, tyrosine, choline, and creatine monohydrate do not [179]. In CVD, it is not clear that multivitamin supplements have a benefit in primary cardiovascular prevention, but others such as carnitine, arginine, and coenzyme Q10 could be beneficial according to preclinical and observational studies [180].

### 2.4. Microbiota

The gut microbiota is an important regulator of the host metabolism. Recent studies have suggested that gut bacteria play a fundamental role in diseases such as obesity, diabetes, and CVD. Data are accumulating in both animal models and humans, suggesting that obesity and T2DM are associated with a profound dysbiosis [181].

Gut microbiota-produced metabolites, such as short-chain fatty acids, amino acid derivatives, and secondary bile acids, participate in glucose homeostasis. A healthy gut microbiota plays a role in health, but imbalances can become pathological, increasing inflammation, and contributing to metabolic dysfunction. Diet plays a significant role in shaping the composition and function of the microbiota. Eating patterns high in fruits, vegetables, whole grains, and legumes promote the abundance of healthier bacteria that produce short-chain fatty acids and other health-promoting metabolites. Jardine (2016) reviewed the functions of the microbiota, how it is formed, and nutritional strategies to improve gut health [182].

## 3. Dietary Patterns for the Management of Patients with CVD and DM

Different diets have been tested in DM and CVD: The Mediterranean (MedDiet), vegan, vegetarian, Korean, paleolithic, ketogenic, and the Dietary Approaches to Stop Hypertension (DASH) dietary pattern. The different types of food in these diets according to the calories ingested (high, moderate, and low intakes) are shown in Table 1.

The MedDiet is amply described and mainly consists of the high intake of vegetables, fruit, legumes, grains, nuts, fish, and water; a moderate intake of red wine, eggs, virgin olive oil, and dairy products; and a low intake of red and processed meats, sweets, and sugary drinks [183,184]. Although MedDiet mechanisms still need to be completely elucidated, it is known that they involve a reduction in both inflammatory and oxidative stress markers, atheroprotective and antithrombotic properties, lipid-lowering effects, increased insulin sensitivity and endothelial functions, protection against platelet aggregation, modification of hormones and growth factors that participate in the pathogenesis of cancer, inhibition of nutrient sensing pathways, and the production of metabolites via gut microbiota to influence metabolic health [183,185]. For these reasons, the MedDiet has been considered an advisable lifestyle pattern by some authors [186,187].

All the beneficial functions mentioned above have led the MedDiet to be recommended as a dietary approach. In addition, the MedDiet has been associated with a more favorable cardiovascular risk profile and better glucose control in T2DM subjects through the fiber intake, wholegrain cereals, and low GI foods such as legumes, fruit, and vegetables, as well as the substitution of saturated fats by monounsaturated fats [188]. Similarly, the MedDiet also reduces the risk of overall mortality, CVD, CHD, myocardial infarction, and T2DM, among other diseases, due to the effects of many nutrients, including fiber, phytosterols, polyphenols, MUFAs, PUFAs, vitamins, and minerals [184,189]. In addition, MedDiet includes a low-to-moderate consumption of red wine and virgin olive oil, which have been reported to prevent CVD, T2DM, and obesity [183]. However, a meta-analysis carried out by Rees et al. (2019), which included more than 12,000 participants, cautiously concluded that the MedDiet effects on clinical endpoints and CVD are still uncertain, since they found high heterogeneity (e.g., participants, nature and duration of intervention, or comparison groups) and an unclear risk of bias [190].

The vegan diet contains only plant foods, with no products of animal origin including eggs and dairy products. However, a vegetarian diet does include those animal-derived foods [191]. Vegan and vegetarian diet pyramids are very similar. They contain a high intake of fruit and vegetables, moderate intake of whole-grain food, nuts, and seeds, and a low intake of herbs, spices, and plant oils. The vegetarian diet also includes a very low intake of eggs and dairy products. Randomized controlled trials, reviews, and meta-analyses concluded that plant-based diets are an effective strategy for the treatment of cardio-metabolic diseases since they decrease all-cause mortality and the risk of obesity, T2DM, CHD, atherosclerosis, blood lipids, and blood pressure, as well as improving both weight and glycemic control [191,192,193,194,195].

The Dietary Approaches to Stop Hypertension (DASH) dietary pattern prioritizes fruit, vegetables, fat-free and low-fat dairy products, whole grains, nuts, and legumes, and limits total and saturated fats, cholesterol, red and processed meats, sweets, and added sugars [196]. The DASH diet is highly recommended to improve cardiometabolic health in patients with diabetes [197]. This diet demonstrated significant drops in HbA1c and LDL-cholesterol concentrations [196], and in systolic and diastolic blood pressure [198,199], and was effective in preventing the development of hypertension in those patients [200]. The DASH diet, accompanied by a reduction of sodium intake, was significantly associated with lower levels of blood pressure irrespective of race, sex, and the presence of hypertension, as well as the reduction of systolic blood pressure compared with the control DASH diet, with high sodium levels [201]. Furthermore, the DASH diet lowered CRP levels in T2DM subjects, thus reducing oxidative stress and inflammation [202].

The Korean diet is characterized by a high intake of grains (rice, bread and noodles), moderate intake of animal and plant foods (meat, fish, dairy products, fruit and vegetables) and low intake of oils and sugars [203]. The Korean diet has been associated with an improvement in the risk of CVD, total and LDL-cholesterol [204], and with a lower risk of hypertension and the metabolic syndrome in women, and a lower risk of hypertension and hypertriglyceridemia in men [205]. This type of diet also has downregulated plasma micro-RNA linked to DM and acute coronary syndrome [206]. Furthermore, the addition of almonds raises MUFAs, PUFAs, vitamin E, and dietary fiber in the Korean diet, thereby reducing the risk of CVD [207].

The Paleolithic diet consists of the intake of proteins (e.g., lean meat, fish, and eggs) and food from uncultivated plant sources, and excludes food unavailable before humans started to cultivate plants such as dairy products, grains, legumes, salt, refined sugar, and processed oils. Although no association was observed in postmenopausal women with T2DM between the paleolithic score and the risk of CVD [30], most studies concluded that this diet has benefits for patients with diabetes and an increased cardiovascular risk [208]. In this respect, the paleolithic diet has proved to lower BMI, CRP levels, and systolic and diastolic blood pressure [209] as well as myocardial triglyceride levels [210] and all-cause and cause-specific mortality [211], while improving left ventricle remodeling [208], leptin levels, insulin sensitivity, and glycemic control [212,213].

The ketogenic diet (KCD) is characterized by a high intake of lipids and a low intake of carbohydrates. There are four types of KCD: traditional KCD, in which high-fat foods are consumed instead of high-carbohydrate food; medium-chain triglyceride KCD, characterized by a greater proportion of proteins and carbohydrates; modified Atkins KCD, with no calorie restrictions and a strong fat intake; and low GI KCD, in which only carbohydrates of low GI can be ingested [214]. KCD proved to be beneficial in T2DM patients since it improved glycemic control and weight [215,216,217] and reduced inflammation, thus being associated with a protective effect for atherosclerosis [218]. The flexibility of KCD, which can also have vegetarian or vegan versions, could be a good choice for personalized diets to treat obesity and DM [219]. The contribution of modified total fasting through the use of formula diets has demonstrated its efficacy and safety to achieve significant weight loss in relatively short periods of time, enhancing ketogenesis, and may be useful in cardiovascular emergency situations, especially heart failure refractory to other treatments [220].

## 4. Concluding Remarks

Cardiometabolic diseases are very prevalent and are associated with a high rate of morbidity and mortality. They are promoted by an inflammatory process that induces hyperglycemia, IR, or ID. DM and CVD are closely interrelated and could be improved by dietary approaches that has been successfully tested in other diseases. Moreover, it is worth keeping in mind that dyslipidemia of patient with T2DM is driven by elevated triglycerides that cause low HDL and smaller and more atherogenic LDL particles, most of times with normal or only moderately elevated LDL cholesterol.

The present narrative review clarified current understanding of the role of nutrition in patients with both T2DM and CVD. Some nutrients such as MUFAs, low-GI carbohydrates, plant proteins, and some minerals, microbiota, and vitamins play a key role in the prevention of the diseases by reducing their risks and mortality, whereas SFAs, TFAs, red meat proteins, high-GI carbohydrates, and food additives promote cardiometabolic dysfunction. The role of other nutrients remains unclear since different results have been obtained in meta-analyses, randomized controlled trials, and clinical trials. This may be due to different parameters involved in analyses related to the variability in nutrient dosages and duration as well as the population studied (e.g., age, sex, race, or ethnicity).

Finally, nutritional interventions must continue tailoring diets to improve T2DM and CVD outcomes; however, personal preferences (e.g., tradition, culture, or religion) should be considered. To this end, further studies, guidelines, and dietary recommendations are particularly required in patients with both T2DM and CVD.

## Figures and Tables

**Table 1:** Intakes of different foods depending on the diet.

Intake	High	Moderate	Low
Diet			
Mediterranean	Fruit → Beans Vegetables → Nuts Whole grains → Seeds Fish → Herbs Legumes → Spices	Red wine → Oil Seafood → Eggs Poultry → Dairy products	Meat Sweets Sugary drinks
Vegan	Fruit Vegetables	Whole grains Nuts Seeds	Herbs Spices Plant oils
Vegetarian	Fruit Vegetables	Whole grains Nuts Seeds	Herbs → Dairy products Spices → Eggs Plant oils
DASH	Fruit Vegetables Grains	Low-fat dairy → Beans Seafood → Nuts Poultry → Seeds Lean meat → Oils	Sweets
Paleolithic	Meat → Eggs Fish → Seafood	Fruit Vegetables	Nuts Berries
Korean	Rice → Fruit Noodles → Bread Vegetables → Seeds Whole grains → Nuts Legumes	Fish Shellfish Dairy products	Eggs Poultry Sweets Meat
Ketogenic	Oils → Seeds Butter → Avocados Nuts	Dairy products → Eggs Meat → Fish	Fruit Vegetables Berries
